# Periradicular Tissue Responses to Biologically Active Molecules or MTA When Applied in Furcal Perforation of Dogs' Teeth

**DOI:** 10.1155/2012/257832

**Published:** 2012-01-26

**Authors:** Anna Zairi, Theodoros Lambrianidis, Ourania Pantelidou, Serafim Papadimitriou, Dimitrios Tziafas

**Affiliations:** ^1^Department of Endodontology, School of Dentistry, Aristotle University of Thessaloniki, 21 Smirnis Street, 562 24 Thessaloniki, Greece; ^2^Department of Clinics, School of Veterinary Medicine, Aristotle University of Thessaloniki, Thessaloniki, Greece

## Abstract

The aim of this study was the comparative evaluation of inflammatory reactions and tissue responses to four growth factors, or mineral trioxide aggregate (MTA), or a zinc-oxide-eugenol-based cement (IRM) as controls, when used for the repair of furcal perforations in dogs' teeth. Results showed significantly higher inflammatory cell response in the transforming growth factor**β**1 (TGF**β**1) and zinc-oxide-eugenol-based cement (IRM) groups and higher rates of epithelial proliferation in the TGF**β**1, basic fibroblast growth factor (bFGF), and insulin growth factor-I (IGF-I) groups compared to the MTA. Significantly higher rates of bone formation were found in the control groups compared to the osteogenic protein-1 (OP-1). Significantly higher rates of cementum formation were observed in the IGF-I and bFGF groups compared to the IRM. None of the biologically active molecules can be suggested for repairing furcal perforations, despite the fact that growth factors exerted a clear stimulatory effect on cementum formation and inhibited collagen capsule formation. MTA exhibited better results than the growth factors.

## 1. Introduction

Perforations represent pathologic or iatrogenic communications between the root canal space and the periradicular tissue. Common causes of perforations are resorptive defects, caries, or iatrogenic events that occur during or following an endodontic treatment [1]. Several factors may interfere with the repair of the defect and at the same time affect prognosis [2]. Convincing evidence exists [2], demonstrating that the location of the perforation in relation to the gingival sulcus has a definitive role in the healing outcome. More specifically, a furcation perforation has poor prognosis leading to periradicular breakdown and consequent loss of the periodontal attachment, while frequently results in tooth loss [3, 4]. At the same time, it has been demonstrated that immediate restoration of the perforation prevents bacterial infection of the wound site [2, 5]. Wound healing with reparative mineralized tissue formation represents the optimal end result of a treatment; tissue regeneration depends on several host and treatment factors. One of the reasons why perforations tend to have such poor prognosis may be the fact that none of the materials used can accomplish tissue regeneration at the treated site.

 A plethora of materials has been utilized for the restoration of perforations, including amalgam, gutta-percha, composite resins, glass ionomers, zinc oxide eugenol cements such as IRM and a reinforced zinc oxide cement based on a mixture of eugenol and ethoxy benzoic acid (Super-EBA), and more recently mineral trioxide aggregate (MTA) [6–9]. Although amalgam has been invariably used in restorative dentistry and apical retrofilling techniques for over a century, its safety and integrity have both been questioned in recent years due to the following phenomena associated with its use: release of ions, mercury toxicity, corrosion and electrolysis, marginal leakage, delayed expansion, and tattoo formation [6, 10]. If on the other hand, restoration of perforations could be achieved by using biological factors with proven properties in tissue repair, the above side effects can be eliminated.

 Growth factors are proteins that play a role in regulating a variety of cellular processes such as cell proliferation, differentiation and angiogenesis and are crucial molecules for wound repair and tissue regeneration. Upon binding to their transmembrane receptors, a cascade of intracellular biochemical signals are transmitted. These signals involve tyrosine kinase or serine/threonine kinase phosphorylation pathways and result in the activation or repression of various subsets of genes. Proteins of the transforming growth factor-beta (TGF-*β*) superfamily, which includes bone morphogenetic proteins (BMPs), together with the formerly known as acidic and basic fibroblast growth factors FGF1 and FGF2, have been found to be localized in the bone, cementum, and healing tissues, providing clues of their potential role on these sites. Similar results have been found for the insulin-like growth factors (IGF-I and -II) and cementum-derived growth factor (CGF) [11–13]. Moreover, osteogenic protein-1 (OP-1), which is also known as BMP-7 definitely seems to increase alkaline phosphatase activity in a dose-dependent fashion, while failing to promote periodontal ligament fibroblast mitogenesis [14].

 Application of dentin matrix components and growth factors in deep cavities has stimulated upregulation of biosynthetic activity of primary odontoblasts (reactionary dentin formation). Pulp-capping studies with a broad spectrum of biological agents, including growth factors and extracellular matrix molecules, showed formation of osteodentin and/or tertiary dentinogenesis (reparative dentin formation) [15–20].

Apart from the growth factors mentioned above, mineral trioxide aggregate has been recommended as a root-end filling material, but it has also been used in pulp capping, pulpotomy, apical barrier formation in teeth with open apexes, repair of root perforations, and root canal filling because of its good sealing ability and biocompatibility [21–23].

The aim of the present study was the comparative evaluation of the inflammatory reactions and tissue responses to four different growth factors, MTA and IRM as control, when used for the repair of furcation perforations in dogs' teeth.

## 2. Materials and Methods

Six healthy male Beagle dogs aged 18–30 months with intact dentitions were used. The experimental protocol was conducted in accordance with the ethical guidelines laid down by the Research Committee of Aristotle University of Thessaloniki (European Communities Directive 24 November 1986-86/609/EEC), for the animal care in experimental procedures and approved by the Ethical Committee of the School of Dentistry, Aristotle University of Thessaloniki, Greece. All measures were taken to minimize pain and animal discomfort.

Each animal was pretreated with 1 mg/kg xylazine (Rompun; Bayer, Germany) intramuscularly, and general anaesthesia was induced with an intramuscular injection of 6 mg/kg thiopentone, (Thiopental; Biochemie, Austria). The animals were intubated with a cuffed endotracheal tube, and anaesthesia was maintained under state using halothane (1.5–2.5%) in oxygen, delivered through a semiclosed breathing circuit.

### 2.1. Experimental Procedure

Permanent premolars and molars of both jaws were selected. All teeth were scaled and polished with a rubber cup, cleaned with 5% iodine solution on the day of the operative procedure, to achieve clear visualization of the treatment outcome. Teeth were isolated with cotton rolls, while saliva flow was controlled with high-speed evacuation.

 The cavities were prepared using a tungsten carbide pear-shaped bur (ISO, no. 330 LSS. White, Lakewood, NJ, USA) at ultrahigh speed with copious water spray. Access to the pulp chamber was gained with a sterile water-cooled carbide bur. Pulp tissue was extirpated with Hedstrom files. The root canals were instrumented, irrigated with sodium hypochlorite (1%), dried with sterile paper points, and obturated with thermoplasticized gutta-percha (Obtura, Spartan) and AH26 sealer. The coronal pulp chamber was thoroughly cleaned from all debris with water spray and sterile cotton pellets soaked with saline. A 1.4-mm-diameter perforation was created in the center of the pulp chamber floor of the experimental and positive control teeth using a sterile round bur (ISO size 4) at low speed. The depth of the intentionally created and standardized perforation was 2 mm into the interradicular bone of each selected tooth. This was guided by use of a rubber stopper as a marker on the shank of the bur. Bleeding was controlled with sterile cotton pellets soaked in 2% lidocaine with 1 : 50,000 epinephrine. A new bur was used for each tooth.

 The perforations were washed with sterile saline and dried with cotton pellets, light pressure was applied to control hemorrhage. The perforations were treated immediately with the following lyophilized human recombinant bioactive molecules which were reconstituted in a solubilized basement membrane preparation (BD Biosciences Greece), or MTA (ProRootMTA, Dentsply Tulsa), or zinc and eugenol hard cement IRM (Dentsply, Germany) according to manufacturers' instructions.

- 14 perforations were treated with basement membrane preparations soaked in 25 *μ*L of solution containing 10 mg of OP-1(recombinant human, R&D Systems) per mL PBS (0.1% albumin control solution).
- 10 perforations were treated with basement membrane preparations soaked in 25 *μ*L of solution containing 1 mg of TGF*β*1 (recombinant human, Sigma) per mL PBS (0.1% albumin control solution).
- 10 perforations were treated with basement membrane preparations soaked in 25 *μ*L of solution containing 10 mg of bFGF(recombinant human, R&D Systems) per mL PBS (0.1% albumin control solution).
- 6 perforations were treated with basement membrane preparations soaked in 25 *μ*L of solution containing 10 mg of IGF-I (recombinant human, R&D Systems) per mL PBS (0.1% albumin control solution).

Preparation of solutions containing biologically active materials, establishment of their doses, and quantitative assessment of adsorbed molecules have been performed as described previously by Tziafas et al. [19].

Restorations were performed with amalgam fillings. The tissue responses were assessed at 3 and 8 weeks aftter operation. At the termination of the experimental periods, the animals were sacrificed by using an excess dose of pentobarbital sodium and jaws were removed. Specimens were subsequently created containing the operated teeth and the surrounding tissues. Briefly, teeth were fixed in 10% neutral-buffered formalin solution for two weeks and demineralized using Morse's solution (50% formic acid +20% sodium citrate) for two months. Finally, teeth were embedded in paraffin and serially sectioned at 7 *μ*m thickness. All sections coming through the furcal perforation site were stained either with Mayer's hematoxylin-eosin stain or using modified Brown-Brenn's technique and following a standardized protocol. Brown-Brenn's technique was used in order to examine if there was bacterial presence at the wound site. A number of specimens were stained with Mallori's trichrome to detect newly formed bone matrix.

### 2.2. Histological Assessment

The stereotypic connective tissue reactions and the periodontal-specific reparative tissue response to the combined effect of perforation preparation and restoration were evaluated under microscopy according to the following criteria.

*Inflammatory Cell Response*. Inflammatory cell infiltration of the amputated tissue area was classified as (a) absent, if no inflammatory cells were detected; (b) slight, if a few scattered inflammatory cells were detected; (c) moderate or severe if a large amount of inflammatory cells were present or abscess formation.

*Epithelium Proliferation*. The junctional epithelial responses were classified as (a) absent if no epithelium was detected; (b) partially organized if traces of epithelium were present; (c) organized if presence of continuous epithelium barrier was visible.

*Hard Tissue Resorption*. The changes were classified as “yes” or “no” based on whether there was tissue resorption in both bone and cementum adjacent to the amputated area.

Hard tissue repair. The changes were classified as “yes” or “no” based on whether there was tissue repair in both bone and cementum adjacent to the amputated area.

Comparisons among different treatment groups were achieved using the Kruskal Wallis and Mann-Whitney U nonparametric statistics. A comparison was considered significant at the *P* = 0.05 cutoff level.

## 3. Results

**Figure 1:**
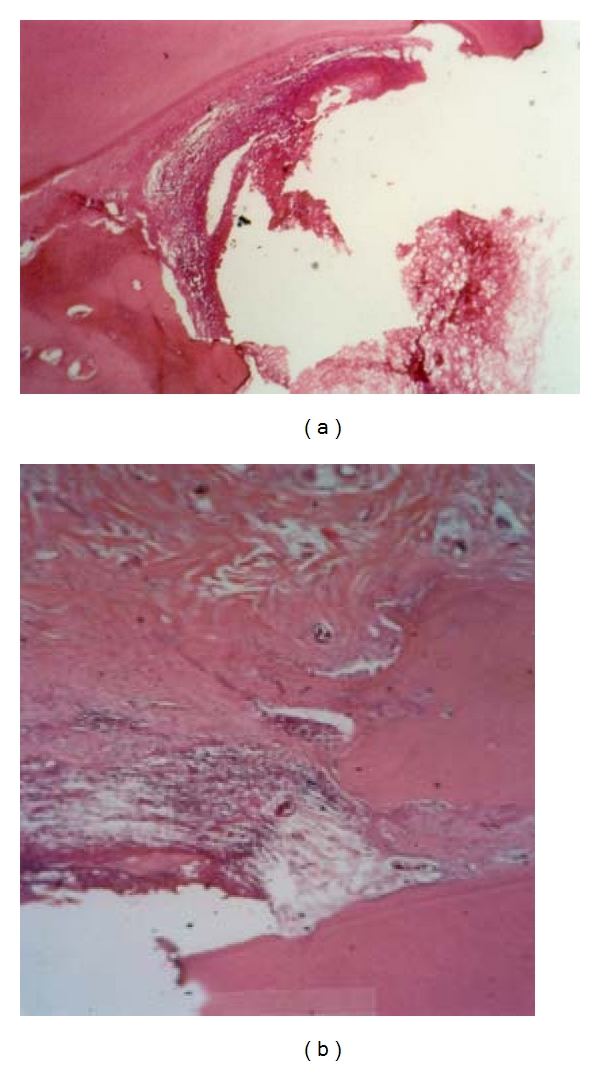
Periodontal tissue response in the dog following a 3- (a) or 8- (b) week application of IRM (control treatment). Note the moderate inflammatory cell infiltration and the superficial necrotic zone. New bone formation and a site of root resorption can be also seen after 8 weeks (magnification ×100).

**Figure 2:**
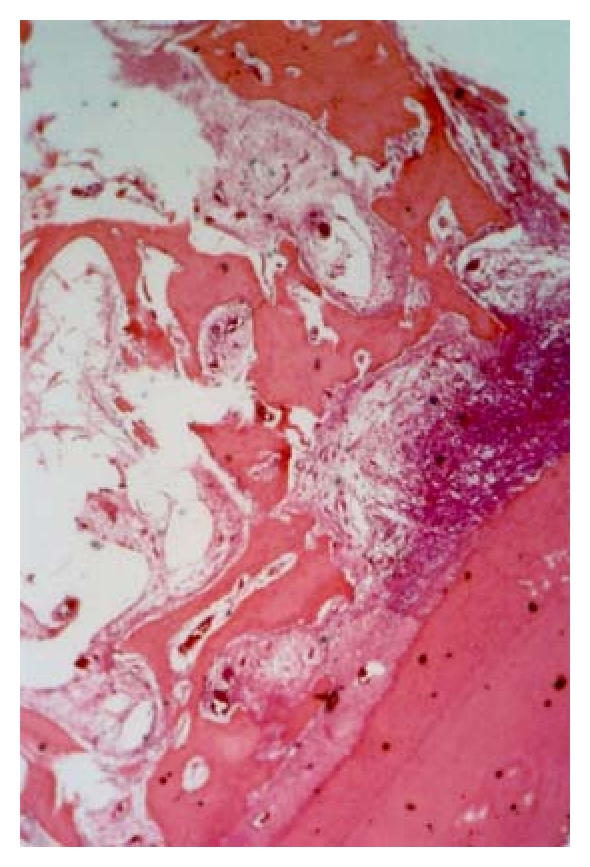
Severe inflammatory infiltration around perforation treated with carrier alone after 3 weeks (magnification ×100).

**Figure 3:**
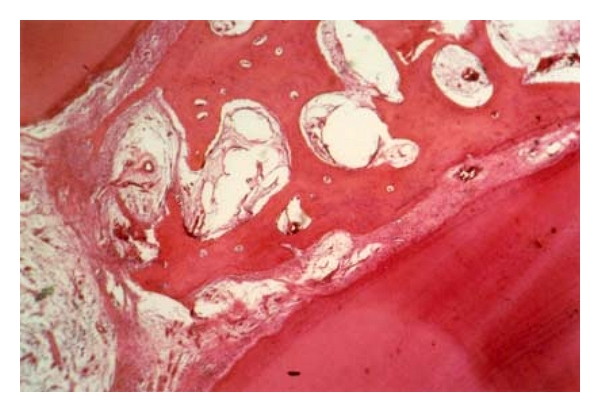
Periodontal tissue response in the dog following an application of IGF-I for 3 weeks. The complete organization of epithelium is evident (magnification ×100).

**Figure 4:**
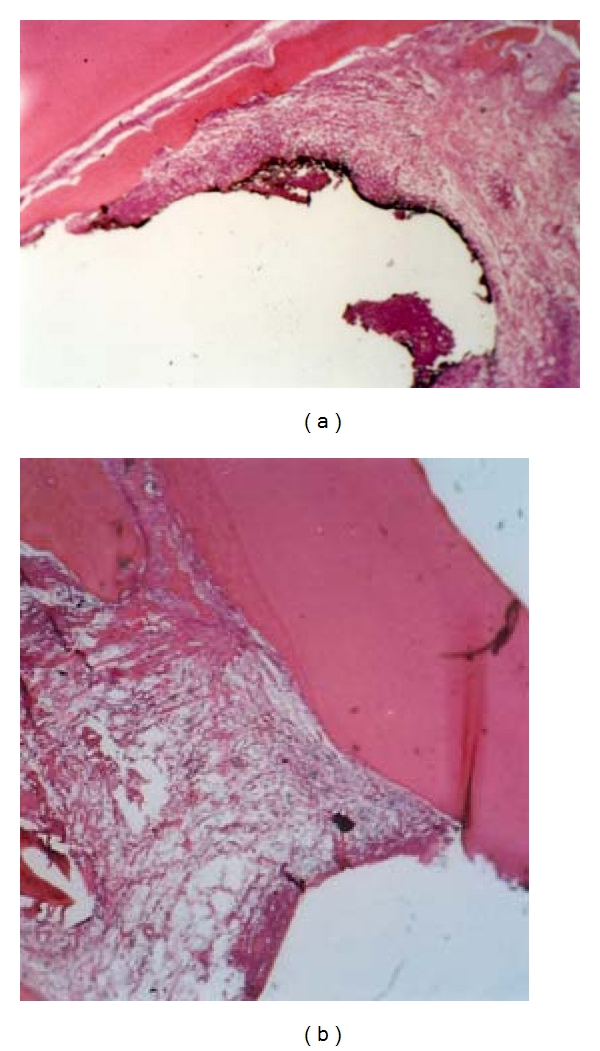
Periodontal tissue response in the dog following an application of bFGF for 8 weeks. Absence of inflammatory infiltration and formation of connective tissue can be seen (magnification ×100).

**Figure 5:**
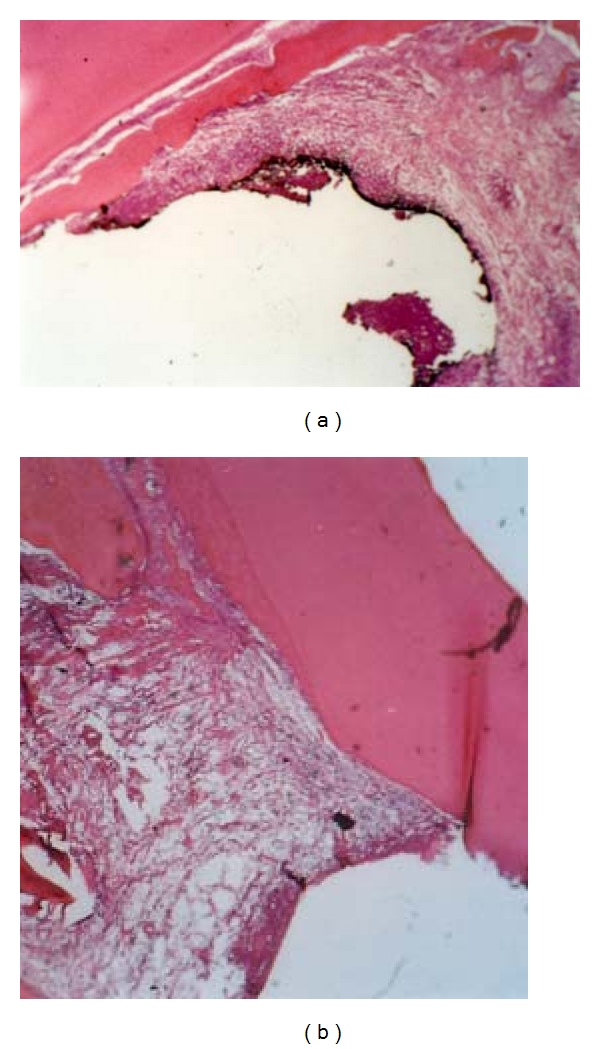
Periodontal tissue response in the dog following a 3- (a) or 8- (b) week application of OP-1. Note absence of inflammatory cell infiltration, traces of new bone formation, and superficial incomplete zone of mineralized matrix (magnification ×100).

**Figure 6:**
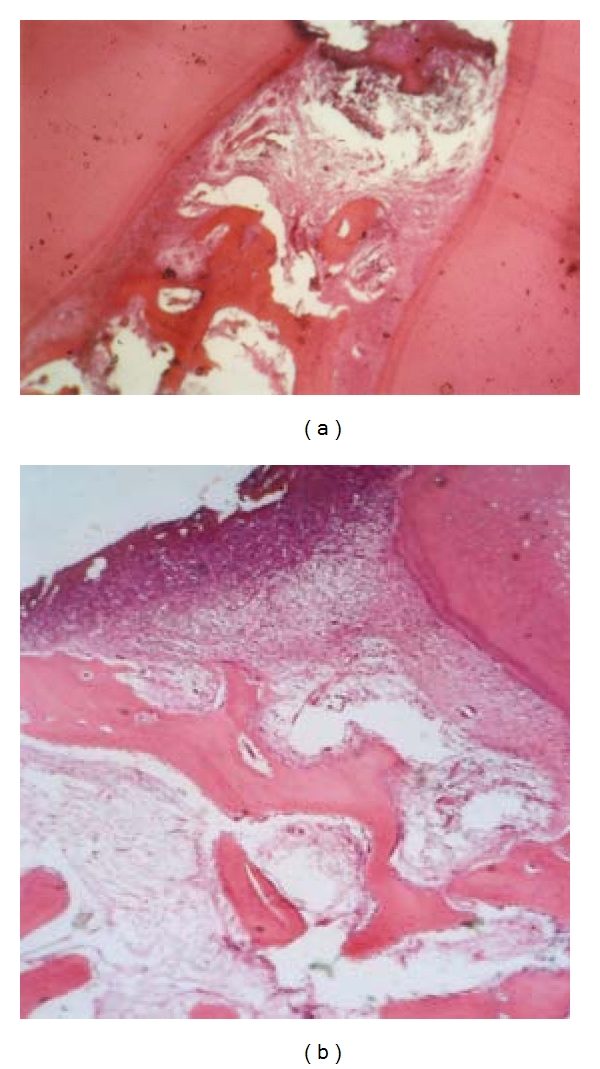
Periodontal tissue response in the dog following a 3- (a) or 8- (b) weeks application of TGF-*β*1. Note the severity of inflammatory cell infiltration and the newly deposited cementum (magnification ×100).

The rate of inflammatory cell filtration, the proliferation of epithelial tissue at the amputated location, resorption rate of bone and cementum and the formation of bone, cementum and connective tissue were compared among the control groups (Figures 1 and 2), the IGF-I group (Figure 3), the bFGF group (Figure 4), the OP-1 group (Figure 5), and the TGF*β*1 group (Figure 6). Results are summarized on Table 1.

Statistical analyses showed the following.

### 3.1. Inflammatory Cell Infiltration

1. There was no statistical difference as far as inflammatory cell infiltration is concerned among the growth factor treated and the IRM (control) groups and the MTA groups at the 3 weeks after operation time point, (Kruskal-Wallis, *X* ^2^(5) = 10.054, *P* = 0.074).
2. A significantly higher inflammatory cell response (Kruskal-Wallis, *P* = 0.007) was observed in the TGF*β*1 group (Mann-Whitney, *P* = 0.003) and the IRM (control) group (Mann-Whitney, *P* = 0.001) compared to the MTA groups at the 8 weeks after operation time point.
3. A significantly higher inflammatory cell response was observed in the TGF*β*1 group (Mann-Whitney, *P* = 0.004) between the 3 weeks and the 8 weeks after operation time point.

### 3.2. Proliferation of Epithelial Tissue

1. Significantly higher rates of epithelial proliferation (Kruskal-Wallis, *P* = 0.007) were observed in the IGF group compared to the MTA group (Mann-Whitney, *P* = 0.005) at the 3 weeks after operation time point.
2. Significantly higher rates of epithelial proliferation (Kruskal-Wallis, *P* < 0.001) were observed in the TGF*β*1 group (Mann-Whitney, *P* = 0.002), in the bFGF group (Mann-Whitney, *P* = 0.002), and in the IGF group (Mann-Whitney, *P* = 0.005) compared to the MTA group at the 8 weeks after operation time point.
3. Significantly higher rates of epithelial proliferation (Kruskal-Wallis, *P* < 0.001) were observed in the TGF*β*1 group (Mann-Whitney, *P* < 0.001), in the bFGF group (Mann-Whitney, *P* = 0.002), and in the IGF group (Mann-Whitney, *P* < 0.001) compared to the MTA group between the 3 weeks and the 8 weeks after operation time point. In addition, significantly higher rates of epithelial proliferation were observed in the IGF group between the 3 weeks and the 8 weeks after operation time point.

### 3.3. Resorption of Bone

Significantly higher rates of bone resorption (Kruskal-Wallis, *P* < 0.001) were observed in the TGF*β*1 group (Mann-Whitney, *P* < 0.001), in the bFGf group (Mann-Whitney, *P* = 0.002), and in the IGF group (Mann-Whitney, *P* < 0.001) between the 3 weeks and the 8 weeks after operation time point. The observation was apparent for both in both time points examined.

### 3.4. Resorption of Cementum

1. A significantly high rate of cementum resorption (Fisher's Exact Test, *P* = 0.004) was observed in the OP-1 group compared to the MTA group (Fisher's Exact Test, *P* = 0.021) and the IRM (control) group (Fisher's Exact Test, *P* = 0.021) at the 3 weeks after operation time point.
2. No difference among the growth factor treated groups of teeth and the IRM treated control group and the MTA group was observed at the 8-weeks post-operation time point.
3. A significantly high rate of cementum resorption (Fisher's Exact Test, *P* = 0.004) was observed in the OP-1 group between the 3 weeks and the 8 weeks after operation time point.

### 3.5. Formation of Bone

1. Significantly higher rates (Fisher's Exact Test, *P* < 0.001) of bone formation were observed in the MTA group (Fisher's Exact Test, *P* = 0.001) and the bFGF group (Fisher's Exact Test, *P* = 0.003) compared to the IRM (control) group at the 3 weeks after operation time point. In addition, significantly higher rates of bone formation were observed in the MTA group (Fisher's Exact Test, *P* = 0.002) compared to the TGF*β*1 group at the 3 weeks after operation time point.
2. Significantly higher rates (Fisher's Exact Test, *P* < 0.001) of bone formation were observed in the MTA group (Fisher's Exact Test, *P* = 0.001), the bFGF group (Fisher's Exact Test, *P* = 0.001), and the TGF*β*1 group (Fisher's Exact Test, *P* = 0.001) compared to the IRM (control) group at the 8 weeks after operation time point.
3. Significantly higher rates (Fisher's Exact Test, *P* < 0.001) of bone formation were observed in the MTA group (Fisher's Exact Test, *P* < 0.001), the bFGF group (Fisher's Exact Test, *P* < 0.001), and the OP-1 group (Fisher's Exact Test, *P* = 0.001) compared to the IRM (control) group between the 3 weeks and the 8 weeks after operation time point.

### 3.6. Formation of Cementum

1. No difference among the growth-factor-treated groups of teeth and the IRM treated control group and the MTA group was observed concerning the cementum formation at the 3 weeks after operation time point (Fisher's Exact Test, *P* > 0.05).
2. Significantly higher rates (Fisher's Exact Test, *P* = 0.001) of cementum formation were observed in the the bFGF group (Fisher's Exact Test, *P* = 0.001) compared to the IRM (control) group at the 8-weeks post-operation time point.
3. Significantly higher rates (Fisher's Exact Test, *P* = 0.001) of cementum formation were observed in the IGF group (Fisher's Exact Test, *P* < 0.001) and the bFGF group (Fisher's Exact Test, *P* = 0.002) compared to the IRM (control) group between the 3 weeks and the 8 weeks after operation time point.

## 4. Discussion

Until today, and to the best of our knowledge, there are no research studies available that evaluated the role of growth factors in healing furcal perforations and included a control group except from one [24]. Kim et al. investigated the effects of repair of apical perforations after applying calcium hydroxide containing growth factors to the lesions. 

As it was shown in our results, successful treatment of furcation perforations can be very difficult. They often result in secondary periodontal involvement with eventual tooth loss [25] and the poor prognosis associated with the treatment, often represents a challenge when evaluating repair materials. 

In this study all perforation defects and the entire access cavities were filled with amalgam because of its durability, ease of manipulation, and good clinical performance. Weldon et al. [9] showed that filling the furcation perforation and access cavity with the same material produced less leakage and therefore prognosis might be improved. Premolars and molars have to bear great stresses from chewing and grinding, and an amalgam-filled molar will resist cracking or breaking. Besides, amalgam is well-tolerated by connective tissue and bone. 

Compared to other research studies that have not examined an agent with reported effects on wound healing, when evaluating several agents, this study examined a number of growth factors for their potential to repair perforations comparing data also for MTA. The lack of such a control group has resulted in inconclusive and contradictory studies in the past which failed to show a beneficial effect of treatment of the tooth with growth factors.

 At the same time, exogenous application of growth factors, which has been widely used in other fields, is a rapidly developing area of dental research. Its basic aim is to regenerate all oral structures, including teeth and periodontal ligament. OP-1, TGF-*β*1, IGF1, and FGF2 were used in our study due to their involvement in oral facial tissue development and regeneration [12–14, 18–20, 22, 26–29]. Especially, OP-1 has well-documented osteoinducting properties by recruiting mononuclear phagocytes to different situations of craniofacial complex [30]. Furthermore, it has demonstrated potent effects on the stimulation of periodontal wound healing including new bone and cementum [12].

BMPs and TGF-*β*1 have been demonstrated to participate in the processes of both reactionary and reparative dentinogenesis [19, 20, 31]. In vitro experiments and genetic studies in animals have also demonstrated that TGF*β*1 is a very important factor involved in dental repair events [31]. FGF exhibits potent angiogenic activity [32] and has demonstrated its ability to induce the growth of immature periodontal ligament cells [29].

IGF-1 regulates DNA and protein synthesis in periodontal ligament fibroblasts in vitro and enhances soft-tissue wound healing in vivo in human tissues [33, 34]. Furthermore, it has anabolic effects when administered to hypophysectomized rats and may be a locally acting mediator of pituitary hormone actions [35].

In comparison to the growth factors, MTA showed better results, which can be attributed to its ability to create an ideal environment for healing [21]. Andelin et al. [36] investigated the presence of dentin sialoprotein (DSP) after pulp capping with either MTA or OP-1. Their results revealed more staining for DSP in the calcified bridge of teeth that were capped with MTA than with OP-1. On the basis of the DSP-positive staining of MTA-capped pulps, the authors concluded that hard tissue produced by MTA has a closer similarity to dentin. Kuratate et al. [37] showed upregulation of osteopontin, nestin, and 5-bromo-2-dexyuridine assay (BrdU) immunopositive cells after pulp capping with white MTA (tooth colored) in rats, but the mechanism of action responsible for the bioactivity of MTA requires further investigation. However, it should be declared that MTA appears not only to demonstrate acceptable biocompatible behavior, but also exhibits acceptable in vivo biologic performance when used as repair material. It should also be noted that the supported data have been overwhelming from either in vitro or animal studies [22].

Using the canine as an experimental animal for our study has certain advantages: premolars and molars of the dog are large and therefore they offer good accessibility and visibility. However, the relationship between furcation area and bone margin of the dog's tooth is not directly comparable to that of the human tooth [8, 38]. Furcation is often as close as 1-2 mm from the cementoenamel junction in the dog. The furcation lies more deeply within the alveolus in humans while the epithelization and the formation of connective tissue is less common. Thus, any technique shown to produce favorable results in dogs may have a more favorable response in humans, where the distance from the cementoenamel junction to the furcation is greater. Thus, it was not surprising to see epithelial proliferation and connective tissue in cases of tissue inflammation in furcal perforation of dogs' teeth [7, 39].

The present experimental data suggest that the application of growth factors in the furcal perforations resulted in stimulation of bone or cementum in the amputated tissue area, while they exhibit an effect on the formation of connective tissue capsule. Capsule formation was detected in the control teeth treated with IRM. Present observation is in accordance with previous reports on the periodontal tissue reactions to IRM, suggesting that the choice of IRM as an internal quality control was successful. In general, slight-to-moderate cell infiltration was found in control groups.we should note that no new bone or cementum was found in this group of teeth. IRM is traditional zinc-oxide-eugenol cement reinforced with polymethyl-methacrylate to improve its mechanical properties that has been broadly investigated in dental research [40, 41]. We used IRM as a control material due to its known behavior to the lesions therefore, it would set a baseline for any effects observed with the growth factor treatment groups.

A number of critical parameters need to be considered when the proliferative and wound healing effects of a growth factor are being examined: the optimum concentration needs to be determined for the specific tissue where the peptide is to be applied, and the carrier needs to be compatible with the site injected, protein instability needs to be considered.

During this study, we used growth-factor-reduced (GFR) Matrigel Matrix as a carrier. It is a solubilized basement membrane preparation extracted from the Engelbreth-Holm-Swarm (EHS) mouse sarcoma, which contains laminin, collagen type IV, heparan sulfate proteoglycan, entactin, growth factors, and other components. By employing a modified method, a more defined preparation of Matrigel is obtained where the levels of endogenous growth factors, excluding TGF-*β* are greatly reduced. This modified form was used due to its reduced levels of EGF, PDGF, and IGF-1, which have been shown to influence cell behavior. It was chosen because of its ease of use, as it gels rapidly at 22–35°C, when it comes in contact with the animal body in the liquefied phase. 

## 5. Conclusions

In conclusion, perforations located in the furcation still remain a challenging as much as demanding field to investigate, since none of the biologically active materials used in the present study can be suggested for repairing perforations. The fact that growth factors exerted a stimulatory effect on bone and cementum formation in the dog and inhibited collagenous capsule formation suggest that they could potentially be used as a treatment strategy for the most problematic area on the endodontic tissues, the furcal perforations. MTA exhibited better results than the growth factors and besides the fact that it can be easily manipulated, it is highly recommended in repairing perforations. Next interesting step is to examine if the effects of application of growth factor mixture with MTA on tissue healing and regeneration is better than MTA alone.

## Figures and Tables

**Table 1:** Periodontal tissue reactions, including inflammatory cell response, epithelium proliferation, bone and root resorption, and matrix formation, classified according to criteria of histological assessment (see in the text).

Type of treatment	*n*	Inflammatory cell infiltration			Epithelium response			Resorption		Matrix formation		
		No slight-severe moderate			No local complete			Bone cementum		Bone cementum soft tissue		
IRM	7/7	0/0	5/6	2/1	7/4	0/2	0/1	0/0	0/0	0/0	0/0	4/5
MTA	6/6	4/5	1/1	1/0	6/6	0/0	0/0	0/0	0/0	6/6	0/2	3/7
OP-1	7/7	0/2	5/4	2/1	2/6	4/0	1/1	1/1	5/1	5/4	0/4	0/0
TGF*β*1	5/5	1/0	4/0	0/5	1/0	2/3	2/2	2/0	2/0	0/5	0/4	0/0
bFGF	4/6	2/1	2/1	0/4	4/0	0/3	0/3	0/0	0/0	4/6	0/6	0/0
IGF	3/3	0/0	2/2	1/1	0/0	1/0	2/3	0/0	0/0	2/1	2/3	0/2
Carrier	4/4	0/0	1/1	3/3	2/2	2/2	0/0	0/4	0/4	0/4	0/4	3/4
